# Prevalence and Associated Factors of Mental Distress among Caregivers of Patients with Epilepsy in Ethiopia: A Cross-Sectional Study Design

**DOI:** 10.1155/2018/2819643

**Published:** 2018-09-27

**Authors:** Sofia Seid, Demeke Demilew, Solomon Yimer, Awoke Mihretu

**Affiliations:** ^1^Amanuel Mental Specialized Hospital, Addis Ababa, Ethiopia; ^2^Department of Psychiatry, College of Medicine and Health Sciences, University of Gondar, Gondar, Ethiopia; ^3^Department of Psychiatry, College of Medicine and Health Sciences, Dilla University, Dilla, Ethiopia

## Abstract

**Background:**

Caregiving to individuals with mental illness is a broad responsibility, including not only practical help and care but also emotional support. Cross-sectional studies in different localities suggested a significant burden of mental distress among caregivers of patients with epilepsy, but we are not aware about the condition in Ethiopia. Therefore, the aim of the current study is to assess the prevalence and associated factors of mental distress among caregivers of patients with epilepsy in Ethiopia.

**Methods:**

An institutional based cross-sectional study was conducted in Neuropsychiatric Department of Amanuel Mental Specialized Hospital, Addis Ababa, Ethiopia. Using systematic random sampling technique, 409 caregivers participated in the study. Data was collected by face to face interview using standardized and validated Kessler Psychological Distress Scale (K-10) to assess mental distress. Descriptive, bivariate, and multivariate logistic regression models were used for analysis. Adjusted Odd Ratio (AOR) with 95% Confidence Interval (CI) was used to show the odds, and* P*-value < 0.05 was considered as statistically significant.

**Results:**

The mean age of respondents was 43.3 years with standard deviation of ±11.4 years. Two hundred eighteen (53.3%) of the respondents were male. The prevalence of mental distress was found to be 27.1% with 95% CI [22.6-31.1]. Relationship with patient of being mother [AOR: 5.67, 95% CI: (1.68-13.70)], father [AOR: 4.42, 95% CI: (1.25-12.58)], wife/husband [AOR: 10.59, 95% CI: (2.43-14.19)], and child [AOR: 5.37, 95% CI: (1.27-12.69)]; caring for young person below 20 years of age [AOR: 4.00, 95% CI: (1.43-11.21)]; poor social support [AOR: 7.26, 95% CI: (3.60-14.65)]); and experienced stigma [AOR: 3.03, 95% CI: (1.63-5.66)] were statistically and significantly associated factors of mental distress among caregivers of patients with epilepsy.

**Conclusion and Recommendation:**

We found a lower prevalence of mental distress among caregivers of patients with epilepsy compared to other low- and middle-income settings. Being caring for young patients, being parents to the patient, poor social support, and stigma were statistically significant associated factors of mental distress among caregivers. Therefore, appropriate psychosocial interventions are warranted to be designed and implemented emphasizing the aforementioned associated factors.

## 1. Background

Caregivers are people who take care of others, often parents, spouse, or children with special medical needs or disability and they play an important role in the management of patients with epilepsy [1–3]. Caregiving is a broad responsibility, including not only practical help and care but also emotional support. Care is more often very stressful task that creates social, emotional, behavioral, and financial challenges for the caregivers, making them prone to mental distress like depression, anxiety, and somatic problems [3–5].

Mental illness influences not only the primary individuals having the diagnosis but also families, friends, and important others around them [6]. Mental distress among caregivers manifests itself with different levels of depression, anxiety, mood disturbances including losing of hope, feeling sad, loneliness, isolation, fearfulness, being easily bothered, nervousness, somatic symptoms such as headache, fatigue, and insomnia arising from providing care for the person in problem [1, 7].

Caregiving is a time-consuming responsibility, creating social, emotional, behavioral, and financial problems for the caregivers and causes various limitations on their personal life. Caregivers within the family have often been described as forgotten patients. Caregivers experience psychological distress including mood swing, fatigue, headaches, joint and muscle pains, and marital and family conflicts [1, 3, 4].

Cross-sectional studies suggested that caregivers of patients with epilepsy experience considerable emotional distress [1, 8, 9]. Caregivers who experience high burden are at risk of developing clinical disorders including depression and anxiety [10].

Epilepsy is the most common chronic neurological disorder characterized by recurrent or multiple seizures [11, 12]. It accounts for 1% of the global burden of disease and affects over 65 million people worldwide. The World Health Organization also estimates that 80% of people with epilepsy live in low- and middle-income countries [12]. The incidence and prevalence of epilepsy are thought to be higher in low- or middle-income countries than in high-income countries. About 3-4 million Africans suffer from epilepsy where treatment gap is estimated to be 80% [1, 11].

A range of factors such as giving care for younger patients, being unemployment, staying longer duration of illness, living in rural area, having family history of epilepsy, having low social support, and experiencing stigma were factors associated with mental distress in caregivers of patients with epilepsy [10, 13–16]. Similar studies also showed that epilepsy is associated with stigma both to the patient with epilepsy as well as the patients' family [17]. Despite being aware about the situation of mental distress among caregivers of patients with epilepsy in different settings, unfortunately we do not know about the psychological burden of epileptic caregivers in relation to their caregiving role in Ethiopia. Therefore, this study aims to determine prevalence and factors associated with mental distress among caregivers of patients with epilepsy at Amanuel Mental Specialized Hospital, Addis Ababa, Ethiopia.

## 2. Methods

### 2.1. Study Design and Period

Institutional based cross-sectional study was conducted from May to June 2017.

### 2.2. Study Area

The study was conducted at Amanuel Mental Specialized Hospital, Addis Ababa, Ethiopia. The hospital has about 300 beds that have been serving all types of mental disorder patients and epilepsy. The hospital has 15 case teams, Neuropsychiatric (NEP) Case Team (with 3 outpatient departments) is dedicated to treatment of patients with epilepsy.

### 2.3. Source and Study Population

#### 2.3.1. Source Population

It included all caregivers of patients with epilepsy who had follow-up at Neuropsychiatric Case Team of Amanuel Mental Specialized Hospital.

#### 2.3.2. Study Population

It included Caregivers of patients with epilepsy who had follow-up at Neuropsychiatric Case Team of Amanuel Mental Specialized Hospital during the study periods.

### 2.4. Inclusion and Exclusion Criteria

Caregivers who were 18 years old and above and who had been providing care for more than 6 months for patients diagnosed with epilepsy were included in the study.

Caregivers who have no direct involvement in providing care, those with history of known psychiatric disorder before being a caregiver, and those who are unable to hear or speak were excluded from the study.

### 2.5. Sample Size and Sampling Procedure

#### 2.5.1. Sample Size

The sample size was determined by using single population proportion formula considering the following assumptions, 95% of confidence interval. There was no local or national data on the prevalence of emotional distress among caregivers of patients with epilepsy. Hence prevalence of 50% and marginal error (d) of 5% were used to maximize sample size.

(1) n=Zα/22p1−Pd2n=1.9620.51−0.50.052=384

Considering 10% for nonresponse rate, the sample size was 384+39=423

where

  n is the sample size calculated,
 
Z_*α*/2_ is a standard Z score 1.96 corresponding to 95% confidence level,
  d is absolute precision or tolerable margin of error of 5%.

#### 2.5.2. Sampling Procedure

Participants of this study were selected from Amanuel Mental Specialized Hospital (AMSH), NEP outpatient departments, through systematic random sampling technique. Sampling interval was determined by dividing the average number of estimated caregivers (1,264) who visited during patients' follow-up in one month data collection time based on one week pilot period out of the average 2,767 epileptic follow-up cases seen monthly by total sample size (423). The sampling fraction was 1,264/423 ≈3. The first respondent was selected by lottery method and the next respondent was chosen at regular intervals of every 3rd caregiver of patients with epilepsy.

### 2.6. Data Collection Instruments

This study used semistructured questionnaires to collect data on sociodemographic, behavioral, and other variables. Standardized and validated scales to measure mental distress using Kessler Psychological Distress Scale (K10), the stigma experience scale, the family version for stigma, and Oslo 3-item social support scale (OSS-3) for social support were used. The K10 is a widely used tool to assess mental distress in the preceding one month [18]. Both the 10-item and 6-item versions (K10 and K6) were validated in Ethiopia, with the 10-item version showing superior validity [19] and used in various studies [19, 20]. Each item is rated from 1 to 5, from “none at all” to “all the time”. The total score for the 10-item scale is 50, ranging from 10 to 50. Respondents were asked about experiencing symptoms of mental distress over the past 1 month. In this study, a study respondent who scored 20 and above was considered as having mental distress. It has been investigated and confirmed that K10 scale had excellent internal consistency reliability, Cronbach's alpha of 0.93 [18].

The stigma experience scale is a frequency scale based on seven items helping to assess their experience of stigma. From the seven items of stigma experience scale the family version, the first four items were scored on a 5-point scale, that is, “never”, “rarely”, “sometimes”, “often”, and “always”. The remaining three items scored using the response categories (yes, no, unsure). Values are then summed across the seven items. Therefore, using mean score as cut of point (score 18), respondents with score 18 and above were considered as experiencing stigma and those with scores less than 18 considered not stigmatized. The coefficient of reliability for this scale was good (0.76); this proves that the scale achieved an acceptable level of internal consistency [21].

Oslo three-item social support scale (OSS-3) provides a brief measure of social support and functioning. In order to score OSS-3, total scores are calculated by adding up the raw scores for each item. The sum of the raw scores has a range from 3 to 14. A score ranging from 3 to 8 is classified as poor social support, 9-11 considered intermediate social support, and a score of 12 to 14 considered as having strong social support. The internal consistency of OSS-3 was reported with a Cronbach's alpha coefficient of 0.50. The relative low Cronbach's alpha does not necessarily reflect a low reliability but a demonstration of OSS-3 multidimensionality. Therefore, the OSS-3 was found to be a potential measuring scale useful in determining range of social support [22].

### 2.7. Data Collectors

Data collection was administered by two BSc psychiatry nurses and one clinical nurse and supervised by one Master's degree psychiatry professional.

### 2.8. Data Quality Control

Pretest was done among 22 of the respondents at Black Lion Hospital in the Neurology Department that has been providing patients with epilepsy follow-up services five days before the actual data collection time to check for the understandability and reliability of the questionnaires. Two-day training on the questionnaire and ethical issues was given for the supervisor and data collectors. Principal investigator closely supervised the data collection process and made spot-checking and reviewing of the completed questionnaires on daily bases to ensure completeness and consistency of the information collected.

### 2.9. Data Processing and Analysis

The data was entered and cleaned with EpiData 3.1 and analyzed using SPSS version 20. Descriptive statistics were used to summarize the data. Binary logistic regression and multivariate models were implemented to investigate factors associated with mental distress. The strength of association was presented by odds ratio with 95% confidence interval (CI). P-value less than 0.05 was considered as statically significant criterion.

### 2.10. Ethical Consideration

Ethical clearance was obtained from Ethical Review Committee of AMSH and UoG. The purpose of the study was explained to each participant before their participation. Formal permission letters were taken from the administrative department of the hospital, and written consent was taken from the participants' for their voluntary participation. Confidentiality is maintained by omitting personal identification.

## 3. Results

A total of 409 caregivers participated in the study with a response rate of 96.7%.

### 3.1. Sociodemographic Characteristics

#### 3.1.1. Caregivers

The mean age of respondents was found to be 43.3 ±11.4 (SD) years. Two hundred eighteen (53.3%) of the respondents were male. Two hundred thirty-six (57.7%) of the respondents were orthodox by religion, one hundred fifty-seven (38.4%) were Amhara followed by Oromo 139 (34.0%) by ethnicity, 96 (23.5%) were mother of the patient, and 376 (91.9%) of the respondents live with the patient (Table 1).

#### 3.1.2. Patients

The median age of the patients was found to be 27 years with interquartile range of 18. The minimum and maximum age was 13 and 80 years, respectively. Two hundred eighteen (53.3%) of patients were male (Table 1).

### 3.2. Clinical Variables

#### 3.2.1. Caregivers

Majority of study participants, 367 (89.7%), had no known medical illnesses. Nineteen (4.6%) of respondents had hypertension and 12 (2.9%) diabetes mellitus (Table 2).

#### 3.2.2. Patients

One hundred fifty-eight (38.6%) patients had less than 5-year duration of illness, and 107 (26.2%) had 5 to 10 years of duration. Two hundred eighteen (53.3%) patients had no experience of seizure in the past three months and only 62 (15.2%) patients had known comorbid illness (Table 2).

#### 3.2.3. Behavioral Variables

Regarding current substance use, majority of caregivers and most patients, that is, 320 (78.2%) and 383 (93.6%), respectively, had no history of substance use (Table 3).

#### 3.2.4. Psychosocial Variables

This study showed that 153 (37.4%) of the caregivers had poor social support. The seven-item stigma experience scale measurement revealed that 79 (19.3%) caregivers had experienced stigma (Table 3).

#### 3.2.5. Prevalence of Mental Distress among Caregivers

**Figure 1:**
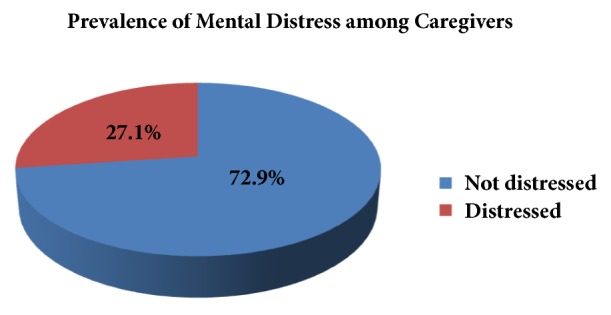
Prevalence of mental distress among caregivers of patients with epilepsy at Amanuel Mental Specialized Hospital, Addis Ababa, Ethiopia, 2017 (n=409).

The overall prevalence of mental distress among caregivers of patients with epilepsy was 27.1%. Among caregivers with mental distress, 65 (58.6%) were females (Figure 1).

### 3.3. Factors Associated with Mental Distress among Caregivers of Patients with Epilepsy

#### 3.3.1. Bivariate Analysis

In the bivariate analysis variables including age, sex, employment, and marital status of the caregivers, relationship with patient, living with patient, social support, and stigma had fulfilled the minimum requirement of p value less than 0.2. Similarly, age, seizure experience, and comorbid medical illness of the patients were found with p value less than 0.2. Therefore, these variables were further entered and analyzed using multivariate analysis.

#### 3.3.2. Multivariate Analysis

In the multivariate analysis variables including age of the patient below 20 years old, relationship with patient (being wife or husband, mother, father, and child), poor social support, and stigma were significantly associated with mental distress among caregivers of patients with epilepsy.

Caring of young patients below 20 years of age was found significantly associated with mental distress among caregivers of patients with epilepsy. Caring of young patients below 20 years of age was about 4 times more likely distressing compared to caring patients with 40 years and above [AOR: 4.00, 95% CI: (1.43-11.21)].

Being mother, father, wife/husband, and child was about 5.6 times, 4.4 times, 10.6 times, and 5.3 times more likely to develop mental distress compared to other relationships such as uncle, aunt, friend, and grandmother/father [AOR: 5.67, 95% CI: (1.68-13.70)], [AOR: 4.42, 95% CI: (1.25-12.58)], [AOR: 10.59, 95% CI: (2.43-14.19)], and [AOR: 5.37, 95% CI: (1.27-12.69)], respectively.

Social support was also found significantly associated with mental distress among caregivers of patients with epilepsy. Caregivers with reported poor social support were about 7 times more likely distressed compared to those with strong social support [AOR: 7.26, 95% CI: (3.60-14.65)].

Experienced stigma among caregivers of patients with epilepsy was also found significantly associated with mental distress. Caregivers with reported experienced stigma were about 3 times more likely distressed compared to caregivers not stigmatized [AOR: 3.03, 95% CI: (1.63-5.66)] (Table 4).

## 4. Discussion

The overall prevalence of mental distress among caregivers of patients with epilepsy in the current study was 27.1% with 95% CI [22.6-31.1]. The prevalence of this study is slightly lower than studies done in Katsina (65.7%), Kaduna (52%), Lagos (39.6%), Pakistan (49%), Western Ontario (50%), and India (52%)[1, 3, 10, 23, 24].

Methodological difference, especially the sensitivity difference of the different screening tools and difference in clinical and other psychosocial factors, of the study participants could be responsible for the discrepancies of the findings. Few of the studies used Burden Assessment Scale and Hospital Anxiety-Depression Scale (HADS) to measure different constructs of mental distress [1, 3, 10]. The prevalence of mental distress in this study is also lower than studies done among caregivers of patients with severe mental illness in general (56.67%) and schizophrenia (48%) in particular in Ethiopia [25, 26]. This could be due to the difference in the challenge of giving care for different mental disorders. In addition, we used Kessler Psychological Distress Scale (K10) where the others used Self-Reported Questionnaire (SRQ 20) for screening.

Regarding associated factors, being mother and father of the patients with epilepsy was about 5.6 times [AOR: 5.67, 95% CI: (1.68-13.70)] and 4.4 times [AOR: 4.42, 95% CI: (1.25-12.58)] more likely to be mentally distressing, respectively, as compared with other caregivers. This is also supported by a study done in India reporting that joint family type and relationship with patients were significantly associated among caregivers of patients with epilepsy [13]. Caring of young patients (13-19 year of age) was 4 times more likely to be distressing compared to caring of patients with 40-year and above age group [AOR: 4.00, 95% CI: (1.43-11.21)].This finding is in line with other studies in different settings including Kaduna-Nigeria, Zambia, America, and Pakistan [10, 13, 27–30]. Caregivers with poor social support were found 7 times more likely to be mentally distressed [AOR= 7.26, 95% CI: (3.60-14.65)] compared to respondents with strong social support.

Lower levels of anxiety among caregivers of younger disable people were also observed in another study. A study conducted in Italy on 342 workers of a cooperative for in-house, outpatient, and scholar social care also showed that those who were working with younger disabled people had lower levels of job stress than those caring for older people. In this study, the symptoms from the low back were significantly related to psychological demands and depression score; symptoms from the upper back were related to age, anxiety, and depression; symptoms from the neck were related to psychological demands, authority over decisions, gender, and anxiety [31].

This finding is also consistent with other studies conducted among caregivers in Ethiopia and India [14, 16, 25]. A study done among caregivers of patients with schizophrenia in India showed that social support has been shown to serve as a buffer against the negative effects that are associated with family caregiving [14]. Stigma was also another significant associated factor. Caregivers involved in this study were found 3 times more likely to be distressed [AOR: 3.03, 95% CI: (1.63-5.66)] compared to those not stigmatized, which is similar to other mental disorders and settings [25, 32–34].

This study indicated that variables such as age, sex, marital status, unemployment, use of substance, living with patient, residence, and known medical illness of the caregivers were not associated with mental distress. Similarly, comorbid medical condition, duration of illness, substance use, and seizure experience of the patient had no association with mental distress. The major limitations of the current study were as follows: The temporal relationship between risk factors might not be addressed since we employ a cross-sectional research design. Recall bias among respondents may also over- or underestimate the result.

## 5. Conclusion

In this study prevalence of mental distress among caregivers of patients with epilepsy was found to be lower than existing studies in different settings. Relationship with the patient (being wife or husband, mother, father, and child), caring of young patients below 20 years old, poor social support, and stigma were found to be significantly associated with mental distress among caregivers of patients with epilepsy. Therefore, appropriate psychosocial interventions are warranted to be designed and implemented emphasizing the aforementioned associated factors.

## Figures and Tables

**Table 1:** Socio-demographic characteristics of caregivers of patients with epilepsy at Amanuel Mental Specialized Hospital, Addis Ababa, Ethiopia, 2017 (n=409).

**Variables**	**Categories**	**Frequency**	**Percentage (**%**)**
**Age (years)**	18-24	33	8.1
	25-34	88	21.5
	35-44	96	23.5
	> 44	192	46.9
**Sex**	Male	218	53.3
	Female	191	46.7
**Religion**	Orthodox	236	57.7
	Muslim	128	31.3
	Protestant	39	9.5
	Catholic	6	1.5
**Ethnicity**	Amhara	157	38.4
	Oromo	139	34.0
	Gurage	80	19.5
	Others	33	8.1
**Marital Status**	Married	268	65.5
	Single	78	19.1
	Divorced/Separated	21	5.1
	Widowed	42	10.3
**Relationship**	Mother	96	23.5
	Father	83	20.3
	Brother/Sister	96	23.5
	Wife/Husband	28	6.8
	Child	62	15.2
	Other caregivers*∗*	44	10.7
**Residence**	Urban	320	78.2
	Rural	89	21.8
**Educational Level**	Can't read and write	96	23.5
	Can read and write	57	13.9
	Primary(1-8 grade)	137	33.5
	Secondary(9-12 grade)	70	17.1
	College and above	49	12.0
**Live with Patient**	Yes	376	91.9
	No	33	8.1
**Employment Status**	Employed	251	61.4
	Not Employed	158	38.6

N.B: ^*∗*^ other caregivers are uncle, aunt, friend, and grandfather/mother.

**Table 2:** Clinical Characteristics as reported by caregivers of patients with epilepsy, Amanuel Mental Specialized Hospital, Addis Ababa, Ethiopia, 2017 (n=409).

**Variables**	**Categories**	**Frequency**	**Percentage (**%**)**
**Known medical illness of caregivers**	Yes	42	10.3
	No	367	89.7
**Co-morbid illness of patient **	Yes	62	15.2
	No	347	84.8
**Duration of illness**			
	Less than 5 years	158	38.6
	5-10 years	107	26.2
	11-19 years	74	18.1
	20-29 years	55	13.4
	30-39 years	15	3.7
**Seizure experience in the past 3 months**	Yes	191	46.7
	No	218	53.3

**Table 3:** Behavioral and psychosocial characteristics of caregivers of patients with epilepsy at Amanuel Mental Specialized Hospital, Addis Ababa, Ethiopia, 2017 (n=409).

**Variables**	**Categories**	**Frequency**	**Percentage (**%**)**
**Current Substance use of caregivers**	Yes	89	21.8
	No	320	78.2
**Social Support**			
	Poor	153	37.4
	Intermediate	122	29.8
	Strong	134	32.8
**Stigma**			
	Yes	79	19.3
	No	330	80.7
**Current Substance use of patients**	Yes	26	6.4
	No	383	93.6

**Table 4:** Factors associated with mental distress among caregivers of patients with epilepsy at Amanuel Mental Specialized Hospital, Addis Ababa, Ethiopia, 2017 (n=409).

**Variables**	**Categories**	**Mental Distress**		**COR(95% CI)**	**AOR(95**%** CI)**
		**Yes**	**No**		
**Age of caregivers (years)**	18-24	3	30	1.00	1.00
	25-34	12	76	1.56 (0.42-5.99)	1.91 (0.41-8.95)
	35-44	25	71	3.52 (0.98-12.55)	3.07 (0.63-13.05)
	> 44	71	121	**5.87 (1.73-12.92)** **∗** **∗**	4.61 (0.89-13.66)
**Sex of caregiver**	Male	46	172	1.00	1.00
	Female	65	126	**1.93 (1.24- 3.00)** **∗** **∗**	1.02 (0.53-1.96)
**Marital Status**	Married	75	193	1.00	1.00
	Single	10	68	1.38 (0.19-2.77)	1.13 (0.42-3.06)
	Divorced/Separated	6	15	1.03 (0.39- 2.75)	0.62 (0.18-2.05)
	Widowed	20	22	**2.34 (1.21- 4.53)** **∗**	1.57 (0.65-3.78)
**Relationship with patient**	Mother	45	51	**8.82 (2.93-14.03)** **∗** **∗**	**5.67 (1.68-13.70)** **∗** **∗**
	Father	25	58	**4.31(1.39-13.34)** **∗**	**4.42 (1.25-12.58)** **∗**
	Brother/Sister	13	83	1.57 (0.48-5.11)	2.22 (0.61-8.12)
	Wife/Husband	11	17	**6.47 (1.80-13.21)** **∗** **∗**	**10.59 (2.43-14.19)** **∗** **∗**
	Child	13	49	2.65 (0.80-8.77)	**5.37 (1.27-12.69)** **∗**
	Other caregiver*s*^*ϕ*^	4	40	1.00	1.00
**Live with Patient**	Yes	106	270	2.20 (0.83-5.84)	1.19 (0.38-3.74)
	No	5	28	1.00	1.00
**Employment Status**	Employed	57	194	1.00	1.00
	Not Employed	54	104	**1.77(1.14-2.75)** **∗**	1.43 (0.77-2.67)
**Age of patients (years)**	13-19	37	36	**4.78 (2.38-9.58)** **∗** **∗** **∗**	**4.00 (1.43-11.21) ** **∗** **∗**
	20-39	57	183	1.45 (0.78-2.64)	1.40 (0.59-3.29)
	>40	17	79	1.00	1.00
**Seizure Experience**	Yes	67	124	**2.14 (1.37-3.33)** **∗** **∗**	1.35 (0.76-2.38)
	No	44	174	1.00	1.00
**Social Support**	Poor	79	74	**9.94 (5.17-19.11)** **∗** **∗** **∗**	**7.26 (3.60-14.65)** **∗** **∗** **∗**
	Intermediate	19	103	1.72 (0.81-3.65)	1.72 (0.78-3.83)
	Strong	13	121	1.00	1.00
**Stigma**	Yes	46	33	**5.68 (3.37-9.58)** **∗** **∗** **∗**	**3.03 (1.63-5.66)** **∗** **∗** **∗**
	No	65	265	1.00	1.00

Note: *∗* p-value < 0.05, *∗∗* p-value <0.01, *∗∗∗*p-value< 0.001 *ϕ* Other caregivers are uncle, aunt, friend, and grandfather/mother.

## Data Availability

The qualitative transcriptions and translations as well as SPSS data are available both in hard copy and electronically.

## Abbreviation

AMSH: Amanuel Mental Specialized Hospital
EpiData: Epidemiological data
ICCMH: Integrated Clinical and Community Mental Health
K 10: Kessler's 10-Item Psychological Distress Scale
MOH: Ministry of Health
NEP: Neuropsychiatry
OSS-3: Oslo 3-Item Social Support Scale
QoL: Quality of Life
SPSS: Statistical Package for Social Science
UoG: University of Gondar
WHO: World Health Organization.
